# Meliponiculture in Quilombola communities of Ipiranga and Gurugi, Paraíba state, Brazil: an ethnoecological approach

**DOI:** 10.1186/1746-4269-10-3

**Published:** 2014-01-10

**Authors:** Roberta Monique Amâncio de Carvalho, Celso Feitosa Martins, José da Silva Mourão

**Affiliations:** 1 Programa de Pós-graduação em Desenvolvimento e Meio Ambiente – PRODEMA, Universidade Federal da Paraíba, João Pessoa, PB 58051-900, Brasil; 2 Departamento de Sistemática e Ecologia, Universidade Federal da Paraíba, João Pessoa, PB 58051-900, Brasil; 3 Departamento de Biologia, Universidade Estadual da Paraíba, Av. das Baraúnas, 351/Campus Universitário, Bodocongó, Campina Grande, PB 58109-753, Brasil

**Keywords:** Reminiscent Communities of Quilombos, Stingless bees, Uruçu boca-de-renda, Ethnoecology

## Abstract

**Background:**

The Quilombola communities of Ipiranga and Gurugi, located in Atlantic Rainforest in Southern of Paraíba state, have stories that are interwoven throughout time. The practice of meliponicultura has been carried out for generations in these social groups and provides an elaborate ecological knowledge based on native stingless bees, the melliferous flora and the management techniques used. The traditional knowledge that Quilombola have of stingless bees is of utmost importance for the establishment of conservation strategies for many species.

**Methods:**

To deepen study concerning the ecological knowledge of the beekeepers, the method of participant observation together with structured and semi-structured interviews was used, as well as the collection of entomological and botanical categories of bees and plants mentioned. With the aim of recording the knowledge related to meliponiculture previously exercised by the residents, the method of the oral story was employed.

**Results and discussion:**

Results show that the informants sampled possess knowledge of twelve categories of stingless bees (Apidae: Meliponini), classified according to morphological, behavioral and ecological characteristics. Their management techniques are represented by the making of traditional *cortiço* and the melliferous flora is composed of many species predominant in the Atlantic Rainforest. From recording the memories and recollections of the individuals, it was observed that an intricate system of beliefs has permeated the keeping of *uruçu* bees (*Melipona scutellaris*) for generations.

**Conclusion:**

According to management techniques used by beekeepers, the keeping of stingless bees in the communities is considered a traditional activity that is embedded within a network of ecological knowledge and beliefs accumulated by generations over time, and is undergoing a process of transformation that provides new meanings to such knowledge, as can be observed in the practices of young people.

## Abstract (pt)

As comunidades quilombolas de Ipiranga e Gurugi, localizadas na Zona da Mata Sul Paraibana, possuem histórias que se entrecruzam ao longo dos tempos. Encontra-se nesses grupos sociais a prática da meliponicultura realizada desde as antigas gerações, fornecendo assim um elaborado conhecimento ecológico, baseado nas abelhas nativas sem ferrão, na flora melífera e nas técnicas de manejo utilizadas. O conhecimento tradicional que os Quilombola possuem sobre as abelhas indígenas sem ferrão é de extrema importância para o estabelecimento de estratégias conservacionistas de diversas espécies.

Para conhecer e aprofundar o estudo sobre o conhecimento ecológico dos meliponicultores foi utilizado o método da observação participante juntamente à realização de entrevistas não estruturadas e semi-estruturadas, além da coleta entomológica e botânica das categorias de abelhas e plantas citadas. Com a intenção de registrar os saberes relacionados à meliponicultura exercida antigamente pelos moradores foi empregado o método da história oral.

**Resultados e discussão**

Os resultados demonstram que no total os entrevistados conhecem uma riqueza de doze categorias de abelhas sem ferrão (Apidae: Meliponini) e classificam-nas de acordo com características morfológicas, comportamentais e ecológicas. Suas técnicas de manejo são representadas pela feitura tradicional do *cortiço* e a flora melífera é composta de variadas espécies predominantes na Mata Atlântica. A partir do registro das memórias e recordações dos indivíduos foi percebido que um intricado sistema de crenças permeia a criação de abelha uruçu (*Melipona scutellaris*) desde gerações passadas.

**Conclusão**

De acordo com as técnicas de manejo utilizadas pelos meliponicultores, a criação de meliponíneos nas comunidades é compreendida como uma atividade de caráter tradicional, que se encontra envolvida numa rede complexa de conhecimentos ecológicos e crenças construídos pelas diferentes gerações ao longo do tempo, e que vêm passando por um processo de transformações e ressignificações dos saberes como pode ser visto, principalmente, nas práticas dos mais jovens.

**Palavras-chave**

Comunidades Remanescentes de Quilombos, Abelhas sem ferrão, Uruçu boca-de-renda e Etnoecologia

## Background

For a long time, human societies have maintained a close relationship with stingless bees, mainly because of their interest in honey, the best-known bee product [1]. Besides their honey and pollen production, nowadays stingless bees have been recognised for their role as the providers of ecosystem services such as pollination of crops and native flora. These social insects occur mainly in Latin America and Africa, particularly in tropical America, and show an expressive diversity and richness of species [2]. In Brazil, Hans Staden was the first to record stingless bees in his book, *Warhaftig Historia,* (1557). Chapter 35 of the book outlines the characteristics of these bees in Brazil, mentioning their typical behaviour, their nesting in hollow trees and the different qualities of honey, as well as describing how the Indians collected honey [3].

With around 600 species stingless bees are representatives of the order Hymenoptera, family Apidae, sub-family Apinae, belonging to the tribe Meliponini [4]. They are also called meliponines (or even native bees and indigenous bees) and belong to a group of bees characterised by the atrophied or absent sting and which, according to a recent list of Camargo and Pedro [5], include 33 genera.

Among the economic, social and cultural relationships between the stingless bees and human societies throughout time, the medicinal use of the resources obtained or derived from them to treat human diseases is notable [6]. The literature contains records concerning the use of honey, pollen, cerumen (wax mixed with plant resins by the bees), larvae, combs, propolis and even batumen to cure several diseases [6-11].

The activity of keeping meliponines, meliponiculture [12], is very common among Brazilian populations and has been performed for centuries by rural populations (mainly from the north and northeast) and traditional communities (such as indigenous people and Quilombolas). Some studies have already been carried out in Brazil, dealing with the relationships among traditional populations and stingless bees [*e.g.*[13-25]. Internationally, some studies in this field can also be cited [*e.g.*[26-31].

The observations and daily practices involved in keeping meliponines for generations, provide such groups with a complex framework for bees, melliferous flora and the ecological relationships between them. Such knowledge is involved in a complex and is integrated by a group of perceptions (*corpus*), productive practices (*praxis*) and system of beliefs (*kosmos*), called a *kosmos-corpus-praxis complex* by Toledo and Barrera-Bassols [32]. The inter-relationship of these aspects rests on knowledge (*corpus*) of human populations and is implemented in the daily practices (*praxis*) and represented in their cultural and symbolic plurality (*kosmos*).

The integration of this complex represents an ethnoecological focus of study, defined as “the field of trans-disciplinary research which studies thoughts, feelings and behaviours which intermediate the interactions among the human populations that have them and the other elements of the ecosystems which include them [33]. The importance of ethnoecological studies, as well as related areas of knowledge such as ethnobiology, ethnozoology and ethnobotany, have been emphasized especially among conservation biologists [34,35].

In the context of ethnoecological research in Brazilian Quilombola communities is necessary to highlight the large representation of these groups in the population, since about over 2,272 Quilombola communities have been certified by the Brazilian government since 1988 [36]. Thus, these groups are characterized and selfrecognize themselves by presenting their own ethnic identity, marked by common ancestry and distinct forms of social and political organization.

The Quilombola communities are also characterised by the existence of a specific territory [37], which is translated as the *lands of common use* and is marked by a diversity of situations where natural resources are appropriated, including usage and property of a private and common character, coupled with ethnic factors, family relationships with cooperation and co-participation [38]. Thus, the *lands of common use* demonstrate that family unity is an essential element, which supports an autonomous production system based on forms of cooperation among different families.

The practice of meliponiculture is associated with the appropriation of natural resources in Quilombola communities and can contribute towards the construction of local sustainability, in view of environmental sustainability. Meliponiculture is an activity that encourages the conservation of stingless bees, ensuring the pollination of native species and plantations, as well as helping to reduce deforestation and damage to the environment.

To investigate the existence of traditional knowledge and pratices of meliponiculture in Quilombola communities, the present study aims to address the following issues: i) identify native bees known to local beekeepers, as well as the characteristics used in bee categories classification, ii) describe the management techniques used by local beekeepers, iii) conduct a survey of melliferous flora, according to the knowledge of local beekeepers, iv) record the traditional beekeeping practices exercised by residents of the communities since ancient times, as well as the symbolic constructions associated with such practices. We hypothesize that: i) the Quilombola communities maintain traditional practices of meliponiculture verbally transmitted through generations. ii) both Quilombola communities share similar practices and traditional knowledge of meliponiculture.

## Methods

### Study area

**Figure 1:**
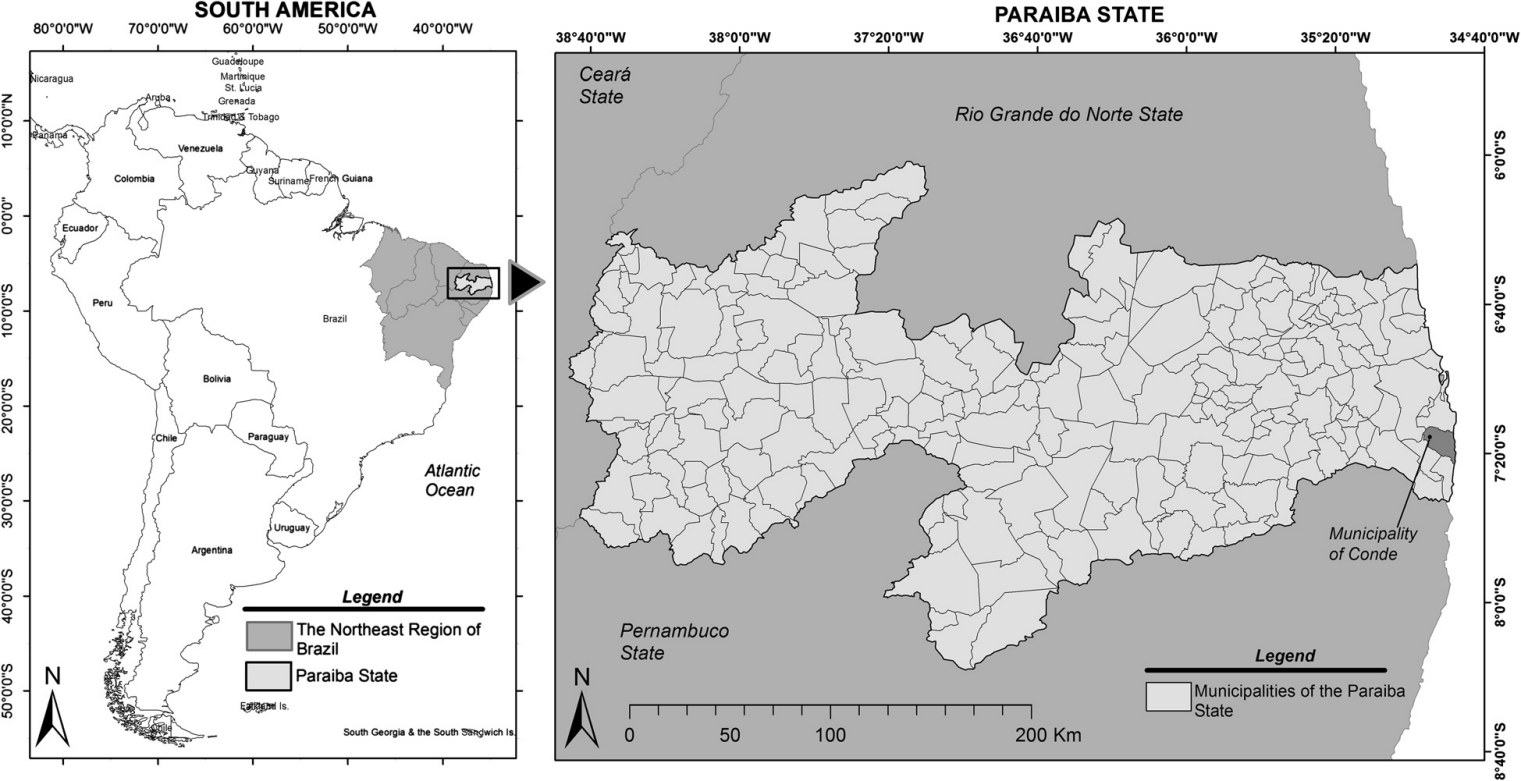
Map showing the municipality of Conde, located in the state of Paraíba.

The Quilombola communities of Ipiranga and Gurugi are located in the municipality of Conde in the state of Paraíba (Figure 1). The municipality of Conde is located to the south of the state capital, João Pessoa (07°15′36″S, 34°54′28″W), in the meso-region of Atlantic Rainforest in Paraíba state. The climate is rainy tropical with a dry summer and the vegetation is predominantly composed by sub-deciduous forest and savannah [39].

**Figure 2:**
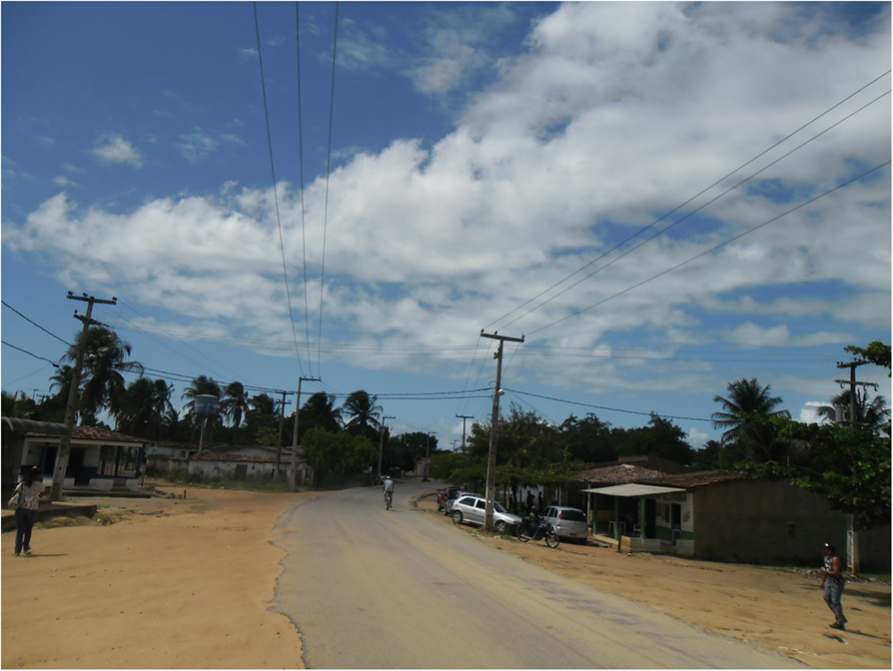
State Highway PB-018 in the centre: on the left is the community of Ipiranga, and on the right, the community of Gurugi.

The communities are approximately 6 km from the centre of Conde and are located on the state highway PB-018 and are adjacent to each other (Figure 2). According to Silva and Dowling [40], a total of 250 families inhabit these two communities and focus their source of income primarily on family farming and fishing, and other extractive activities. Moreover, preserve cultural expressions that are well represented by the dance *coco de roda*.

### Procedures

The research was carried out between May 2011 and November 2012. Initially, the participant observation method was used [41] from visits, participation in meetings and celebrations. This phase was accompanied by informal interviews and a field journal [42]. The choice of informants was performed via the technique of “snow ball” intentional sampling [43], which consisted of an initial meeting with a beekeeper of the region, which led to meetings with others. Following this selection technique, an investigation was undertaken of all families in the communities, which resulted in the recognition of a total of 10 beekeepers (seven in the community of Ipiranga and three in the community of Gurugi), aged between 27 and 87, eight men and two women. Thus, all informants represent the total number of practitioners of this activity among the 250 families.

Non-structured and semi-structured interviews were carried out with the 10 informants [44], and non-structured interviews were performed according to the methodology of data collection [45]. The bee colonies of the informants were then visited and the honey-collecting process was observed, amongst other activities. Among the informants, “native specialists” were chosen according to the criteria of self-recognition and recognition by their own community as being culturally competent [46].

The bee species collected were stored in the Entomology Laboratory of the Federal University of Paraíba (UFPB). The identification of the categories that contained the bees already mentioned but that were not found in the region, was carried out using samples belonging to the collection of the laboratory, which were taken from the field, together with pictures of nests of these species. During the collection of the species from the melliferous flora, a guided tour was performed [47] with one of the research informants and the species collected were identified and deposited in the Herbarium Lauro Pires Xavier of the UFPB.

During the recording of the traditional practices of meliponiculture exercised by the residents of these communities since ancient times, the oral story method was used [48]. Seven informers were chosen for the interviews of oral stories, based on the criterion of a family relationship with old stingless bee keepers in the two communities. Approval for the study was obtained from the Ethics committee of Universidade Estadual da Paraíba and consent was obtained from the informers for the publication of this report and any accompanying images. The permission of the syndicate of Quilombola communities (Associação da Comunidade Negra do Ipiranga, and Associação da Comunidade Negra do Gurugi) was also required to interview the beekepers.

### Data analysis

The data were analysed using an essentially qualitative approach [49]. The field notes were organised as reminders, extensive field notes, and a field journal [44]. The interviews were faithfully transcribed and, where necessary, the consistency and validity of the information collected were checked by the creation of synchronic and diachronic situations [50].

All the data were organised and subsequently selected and condensed as tables and diagrams. Finally, the analysis followed the *emic* and *etic* approach, in which the *etic* mode was considered the way in which the culture members under study perceive, structure, classify and articulate their universe, integrated into the *etic* mode defined as how the researcher perceives the studied culture.

## Results and discussion

### Identification and classification of the bees

All the informants recognised a total of 12 categories of stingless bees (Table 1). The species cited were *Melipona scutellaris* (10 citations); *Melipona subnitida* (10 citations); ““uruçu-boi” (2 citations); *Partamona littoralis* (10 citations); *Plebeia flavocincta* (10 citations); *Frieseomelitta francoi* (10 citations); *Trigona spinipes* (10 citations); *Frieseomelitta dispar* (3 citations); *Scaptotrigona* sp. “abelha-canudo do cano grosso” (2 citations); *Scaptotrigona* sp. “abelha-canudo do cano fino” (2 citations); *Scaptotrigona aff. tubiba* (2 citations); “mumbuca” (2 citations).

**Table 1:** Categories of stingless bees mentioned by the 10 beekeepers from the communities of Ipiranga and Gurugi, state of Paraíba

**Categories**	**Scientific names**
Uruçu boca-de-renda or legitimate uruçu or uruçu	*Melipona scutellaris*
Uruçu-boi	*Non-identified*
Jandaíra or uruçu-mirim	*Melipona subnitida*
Cupira	*Partamona littoralis*
Abelha-mosquito	*Plebeia flavocincta*
Moça-branca or jati	*Frieseomelitta francoi*
Aripuá ou arapuá or abeia-preta	*Trigona spinipes*
Mané-de-abreu	*Frieseomelitta dispar*
Abelha-canudo do cano grosso	*Scaptotrigona sp.*
Abelha-canudo do cano fino	*Scaptotrigona sp.*
Tubiba	*Scaptotrigona aff. tubiba*
Mumbuca	*Non-identified*

The bees were classified according to their behaviour, i.e., whether aggressive or calm, as *mild bee* or *fierce bee*. The category *mild bee* was used to classify *uruçu boca-de-renda* and the *mosquito bee* because they are stingless. Alternatively, *fierce bee* was used to denote specifically *tubiba*, characterised by its aggressive behaviour of biting the beekeeper during honey collection.

Other studies have also recorded the categorisation of stingless bees as *mild* or *fierce*[19,20,24,25]. However, in Léo Neto [25] and Rodrigues [24] the category *fierce bee* was used to designate stinging bees (= *“italian”* bees, africanized *Apis mellifera*).

**Figure 3:**
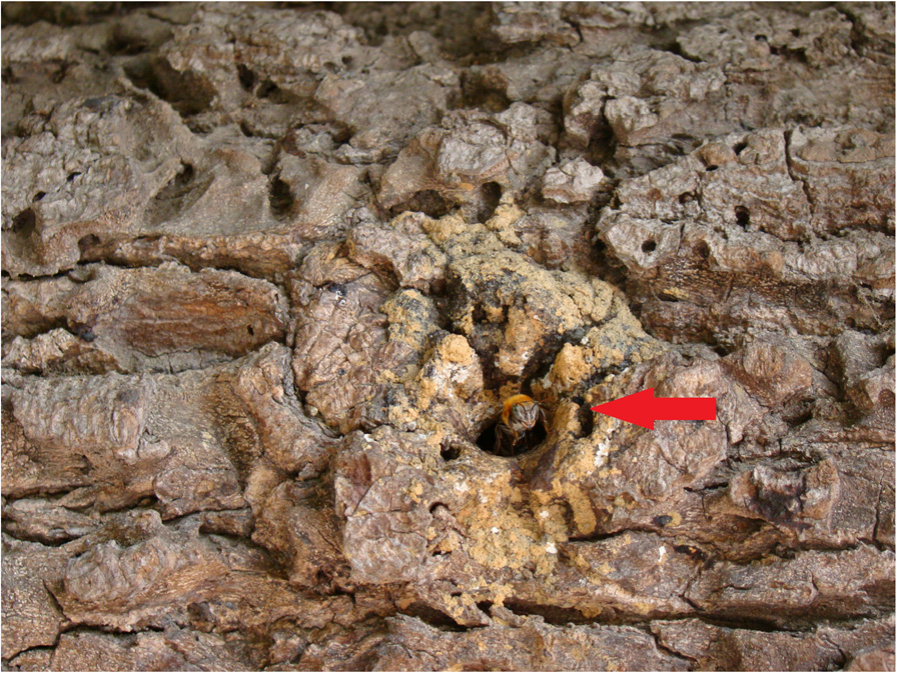
***Guard bee *** **(highlighted) at the entrance to a nest of *uruçu boca-de-renda *** **(Community of Ipiranga, state of Paraíba).**

Regarding the behaviour of the bees within their nest, the informant recognised the category of *queen bee* when they referred to the bee which is the only one in the colony and has a larger size than the other bees of the nest. In addition, other two categories were recognised: *foragers* and *guard bees. Foragers bees* are those that fly into the fields and search for food resources and *guard bees* remain at the entrance of the nest (Figure 3).

The bees were classified by the informants according to morphological characteristics (shape, presence or absence of sting, size, and standard color), behaviour (presence or absence of aggressiveness) and ecological characteristics (nesting places, external and internal characteristics of the nest, dietary habits, characteristics and honey production) (Table 2). Zamudio and Hilgert [29] identifying the elements that the local population from northern Argentina uses to classify the stingless bees, also reported morphological (shape, size and color), behavioral (docile, aggressive or shy) and ecological characteristics (external and internal characteristics of the nest).

**Table 2:** Characteristics used to classify bees by beekeepers from the communities of Ipiranga and Gurugi, state of Paraíba

**General characteristics**	**Specific characteristics**	**Local speech of informants**
Morphological	Shape	“A uruçu é maior, a jandaíra é o mesmo feitio, mas sendo menor”.
		“A italiana, as costa dela aqui é bem cabeludinha. A uruçu não”.
		“The uruçu is larger, jandaíra is the same shape but is smaller”.
		“The back (= thoracic dorsum) of the italian bee is quite hairy. The uruçu is not”.
	Presence or absence of sting	“Ela num tem ferrão não. É a cupira. Abelha cupira”.
		“A uruçu ela não tem ferrão, a europeia ela já tem o ferrão, a abelha aripuá ela também não tem ferrão”.
		“It has not a sting. It is the cupira. Bee cupira”.
		“The uruçu has not a sting, the european (= africanized honeybees) has a sting, the bee aripuá also has no sting”.
	Size	“Tem uma abelha pequenininha que chama abelha-mosquito. Tem a moça-branca, que é outra abelha maiorzinha”.
		“A jati é bem miudinha”.
		“There is a small bee called mosquito bee. There is the moça-branca, which is a little bigger”.
		“The jati is very small”.
	Standard colour	“Se tem uma abelha voando ali e eu to vendo que ela é amarela, de cara eu vou dizer que é uma moça-branca”.
		“A mané-de-abreu é quase que nem mosquito, quase roxinha. A canudo é preta, que nem a tubiba. Uma preta clara sabe?”
		“If there is a bee flying over there and I see it is yellow, I will say it is a moça-branca”.
		“The mané-de-abreu is similar to mosquito, almost purple. The canudo is black, as tubiba. A light black colour you know?”
Behaviour	Presence or absence of aggressiveness	“Cupira é parecida com a aripuá, que a aripuá é outra dessa que pega no cabelo. […] Essa cupira morde pra caramba!”
		“Essa moça-branca ela faz só tocar em você, não morde”.
		“A tubiba era braba que só a gota, quando tirava ela, ela era perigosa”.
		“Cupira is like the aripuá, aripuá are those who pick up the hair. […] The cupira has a powerful bite!”
		“The moça-branca bees just touches you, they do not bite”.
		“The tubiba is very angry when the nest is collected, it is dangerous”.
Ecological	Nesting places	“A uruçu é mais em oco de pau, né? Já a aripuá, ela faz mais em galho de árvore, né? A mosquito é coquinho e pau podre”.
		“Aripuá faz geralmente as casa em cupim, cupinzeiro”.
		“The uruçu makes the nest inside hollow trunks of trees, right? While aripuá nests on tree branchs, right? The mosquito nests inside coconuts and rotten wood”.
		“Aripuá usually makes its ‘house’ (= nest) in termite nest”.
	External characteristics of the nest	“A boca-de-renda você pode olhar ali a boca dela que ela faz uma rendinha na boca, de barro. E a uruçu-boi ela não faz boca de renda”.
		“A tubiba faz a casa e faz o caminho de sair e entrar”.
		“The boca-de-renda you can look its ‘mouth’ (= nest entrance), it makes a lace of clay at the entrance. And the uruçu-boi does not make an entrance with lace”.
		“The tubiba makes the ‘house’ and makes his way to come and go”.
	Internal characteristics of the nest	“A uruçu tira o suco da flor, leva pra lá, lá ela faz as caixinha, tudo redondinho assim com a boquinha aberta”.
		“As bolotinha de mel da abelha-mosquito é quase idêntica as da uruçu, só que a da abelha-mosquito é bem menor, né?”
		“The uruçu takes the juice of the flower (= nectar), takes over there, there it makes the honey pots, all round with a small opening”.
		“The honey pots of the mosquito bee is almost identical to the uruçu, however the honey pots of the mosquito bee is much smaller, right?”
	Dietary habits	“A aripuá ela é uma devoradora dessas florzinha. Ela destrói aquilo ali, as flores, o fruto também, ela destrói muito o fruto da banana, da manga, do caju. A abelha-mosquito ela já é diferente assim, porque ela gosta muito de coisa doce, né? De mel, o mel de qualquer outra abelha ela vai lá, cata, né?”
		“The aripuá is a destroyer of flowers. It destroys the flowers, and the fruits also, it destroys the very fruit of banana, mango, cashew. The mosquito bee is so different, because it really likes sweet things, right? So the honey of any other bee it goes there and gathers, right?”
	Characteristics and honey production	“É o mel melhor que tem é a boca-de-renda, porque a jandaíra ela é uruçu, mas não é o mel bom que nem a boca-de-renda”.
		“A abeia-preta, essa arapuá, faz o mel de todo troço. Agora a uruçu legítima e a jandaíra só faz do suco da flor”.
		“The best honey there is from the boca-de-renda, because jandaira is uruçu, but its honey is not as good as that from the boca-de-renda.”
		“The abeia-preta, arapuá, makes honey from everything. However, the legitimate uruçu and jandaíra only make honey from the juice of flowers”.

However, among the Kayapó Indians in the Amazon [18] and the Atikums in Pernambuco [25], other characteristics little known to the ethnoentomologists were used in the classification of stingless bees, such as differences in flight and the smell that each species exudes.

As recorded by Costa-Neto [19] during study of the Pankararés, in the taxonomic system of the communities studied here, the presence of a prototypical taxon, the *legitimate uruçu* was noted. The informants frequently attribute specific properties to *uruçu boca-de-renda*, in terms of the characteristics of the production and medicinal quality of their honey. According to them, *uruçu* collects resources from specific flowers to produce honey and, for this reason, their honey is medicinal. Thus, it is often called *legitimate uruçu*, compared to *uruçu-mirim* and *uruçu-boi*.

### Products used by the beekeepers

In the communities, honey, cerumen and *saburá* (pollen) are used. As mentioned previously, the honey produced by *uruçu boca-de-renda* is considered the best honey by all informants, because of its medicinal properties. Thus, *uruçu boca-de-renda* is the bee that permeates all meliponiculture in the communities of Ipiranga and Gurugi for generations, with their honey widely being used in the treatment of several diseases throughout the years (Table 3).

**Table 3:** **Medicinal recommendations for the honey from *uruçu boca-de-renda*, according to beekeepers from the communities of Ipiranga and Gurugi, state of Paraíba**

**Indications**	**Application**
Throatache	Eaten
Cough	Eaten
Earache	Massaged with cotton
Oral micoses (“sapinho”)	Massaged with cotton
Hemorrhoid	Directly on the site
Ocular cataracts	Directly on the site
Pterygium (“vilídia”)	Directly on the site
Conjunctivitis	Directly on the site
Stimulating appetite	Eaten
Restorative	Eaten

Oliveira *et al*. [8] describe the use of uruçu honey (*Melipona scutellaris*) for mouth ulcers in children and flu. Moreover, in a review conducted on the use of animal remedies in traditional medicine in Latin America, Alves and Alves [6] also mention the use of uruçu honey in the treatment of coughs, oral fungal infections, eye problems, cataracts and weakness.

Currently, the honey is mostly exchanged among the families rather than being bought and sold. Thus, the communal use of the melliferous resources was a characteristic noted among the residents, since the honey is always given or exchanged for other resources.

When questioned about the procedure carried out by the bees during honey production, there is a consensus among the informants that the process corresponds to the *science* of the bees and it is very difficult to be understood by humans. Oliveira [20] reports that the rubber tappers and the Kaxinawá tribe from the upper Juruá River also do not know about the process of honey production and they state that its method of production is a mystery, which is presented as “the great science of the bees”.

**Figure 4:**
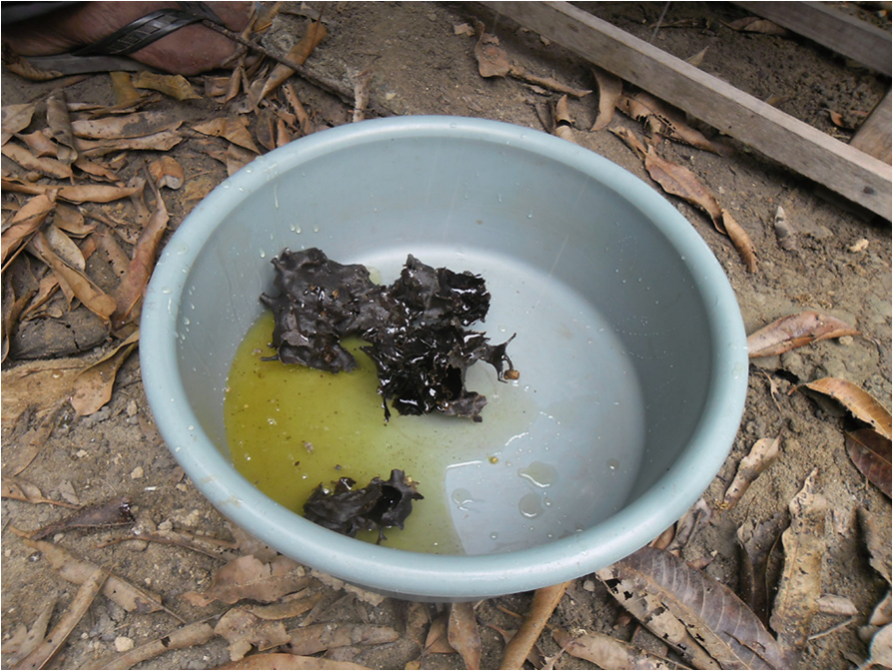
**Pieces of *virgin cerumen *** **removed when the honey is collected (community of Ipiranga, state of Paraíba).**

The main use of cerumen comes from the *virgin cerumen*, which is the cerumen removed from the honey pots when it is collected and put to dry in the sun, without being eaten (Figure 4). The *virgin cerumen* is employed in the smoking of the nests, when the honey is collected, with the aim of calming down the bees and it also has medicinal uses, such as for ear ache, healing wounds and the blockage of airways.

**Figure 5:**
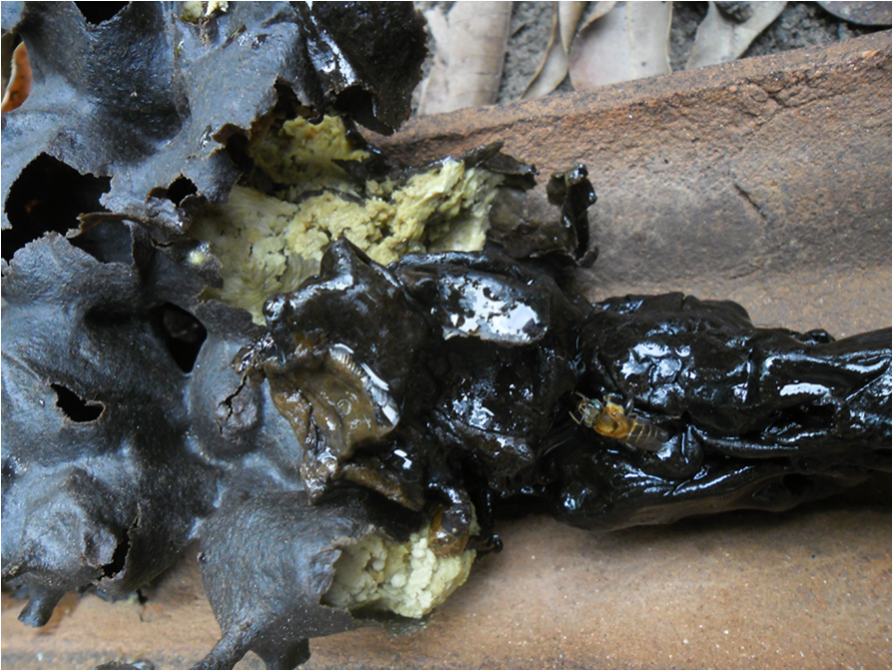
**Pots of *saburá *** **removed when the honey was collected in the community of Ipiranga, state of Paraíba.**

The informants refer to the pollen stored in pots by the bees for their feeding as *saburá*. As well as among the rubber tappers and the Kaxinawás from the upper Juruá River [20], the beekeepers remove the *saburá* from the colonies at the moment of honey collection and throw it away (Figure 5), thus it is not used for any purpose. However, Souto *et al*. [10] describe the use of the *saburá* from *cupira* bees (*Partamona seridoensis*) in ethnoveterinary procedures in northeastern Brazil.

### Keeping the stingless bees

The bees managed are *uruçu boca-de-renda*, *moça-branca*, *mosquito bee* and *jandaíra*.

**Figure 6:**
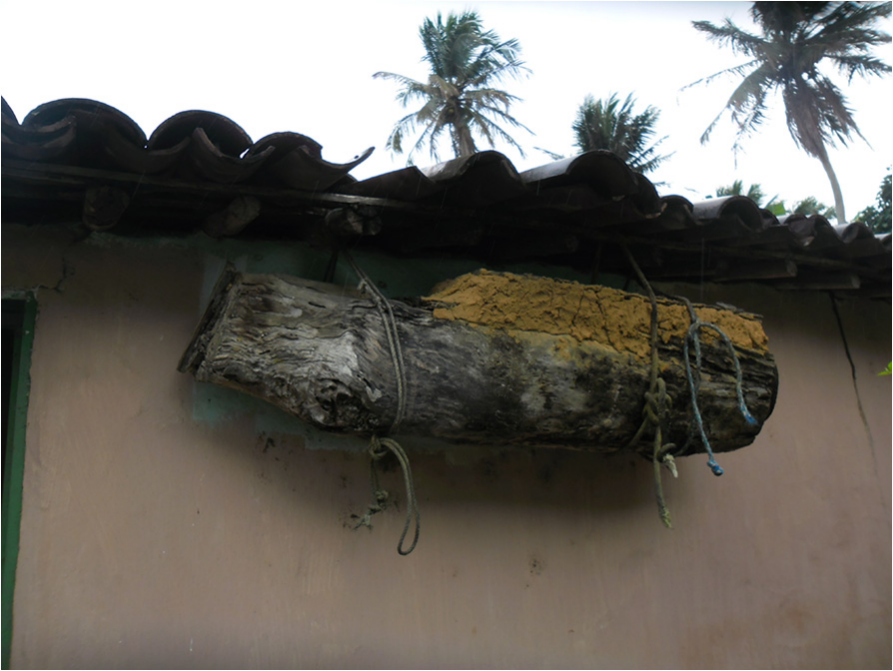
**Example of *cortiço *** **found in the community of Ipiranga, state of Paraíba.**

**Figure 7:**
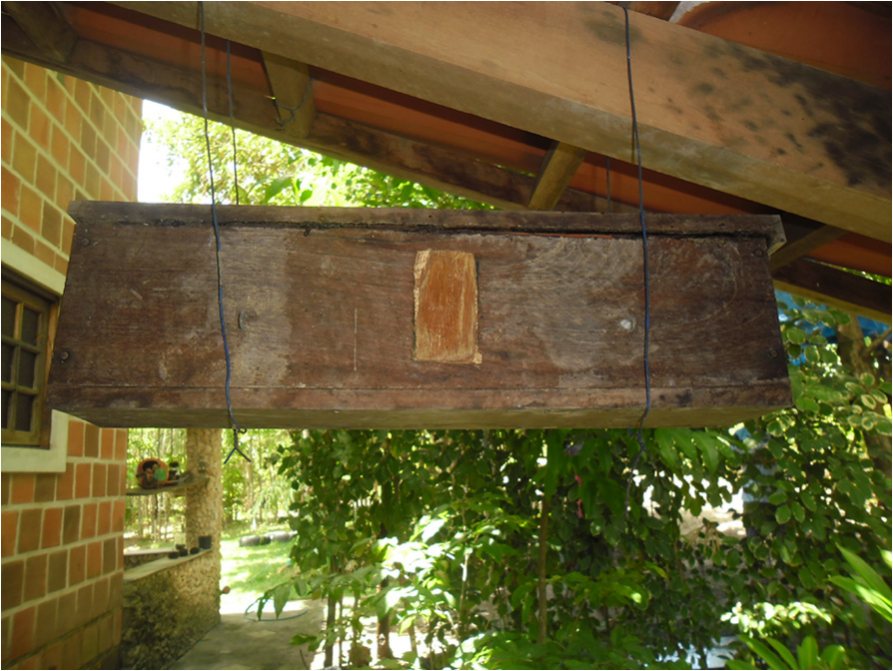
Example of a rustic box from the community of Ipiranga, state of Paraíba.

When *moça-branca*, *mosquito bee* and *jandaíra* are managed, the informants from both communities use rustic boxes, which are bought or made by themselves. However, the bee *uruçu* is more frequently managed being kept by 90% of informants. This bee is managed by both communities via the traditional technique of *cortiço,* which consists of removing hollow trunks of trees in which the nests are located, closing the extremities with clay and transporting them to their houses (Figure 6). However, the practice of keeping bees in *cortiços* is currently being replaced, especially by the younger beekeepers, by rustic boxes. When they were questioned about this change, the younger informants answered that the rustic boxes make bee management easier (Figure 7).

One of the informants, who was characterised for this research as a “native specialist”, was committed to transmitting the *cortiço* technique, by teaching it to his children and grandchildren and resisting the transfer all of his colonies to rustic boxes. According to Giddens [51], the tradition has “guardians”, who are those who identify the details of the traditions via interaction with others of the same age and teach them to the youth. The “tradition guardians”, for Giddens [51], are those who have a connection with the truths that traditions contain or reveal, and these truths are manifested in the interpretations and practices of the “guardians”.

With regard to the artificial division of the colonies, the informants report that this occurs infrequently, only when there is a need to duplicate the original colony to increase honey production. According to them, it is firstly necessary to find a new *cortiço* (or box) that will shelter the new colony that will result from the division and then some honeycombs from the original nest are placed in the new one. This should be placed exactly in the same location as the original nest, which is allocated a new site. Following this, the new nest will receive the *foragers* that return with the food resources collected and, thus, the new colony will be established.

There is no consensus among the informants regarding the frequency of honey collection; some of them collect it every three months, others every six months and others do not have a defined time. However, the general consensus is that the best time to collect honey is during the spring, mainly between September and the middle of January, which is called *flower time*. When the collection is carried out, the honey pots are pierced with a piece of wood and the *cortiço* is inclined to the side which has a small orifice where the honey flows. The honey is then strained through a clean cloth and stored in glass bottles.

According to the informants, the productivity of *uruçu* colonies varies from 4 L to 8 L of honey a year per colony. In contrast, the *moça-branca*, *mosquito* and *jandaíra* colonies produce a lower amount of honey, yielding less than 1 L a year per colony.

**Figure 8:**
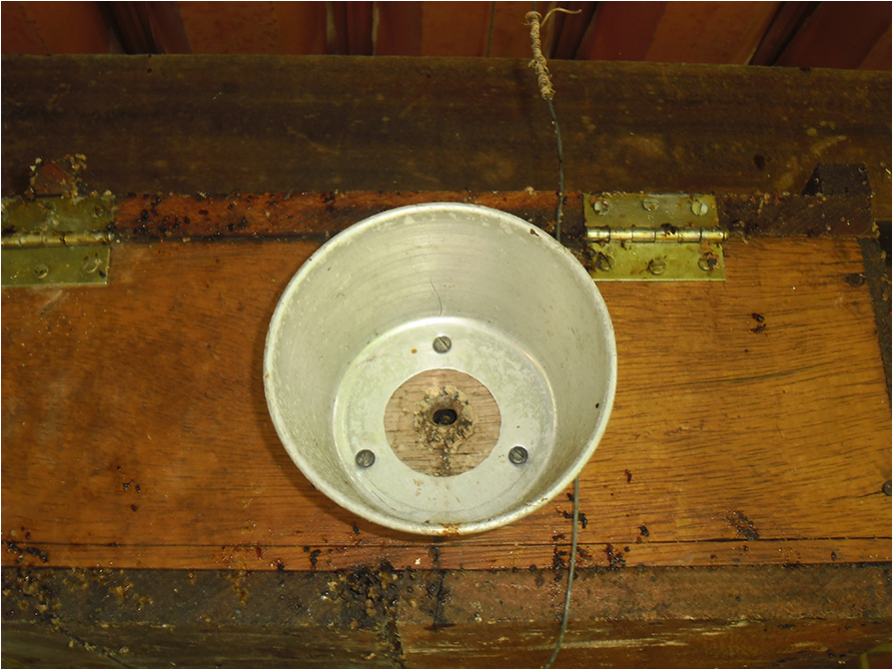
Detail of the aluminum utensil used by some beekeepers to combat lizards (community of Ipiranga, Paraíba).

The most mentioned predator by the informants was the lizard (*Tropidurus hispidus* and *Hemidactylus mabouia*), which remains at the entrance to the *cortiço* or the boxes. Lizard control is performed by either placing a piece of aluminium, a can or a plastic bottle at the entrance of the nest (Figure 8).

### The melliferous flora

**Figure 9:**
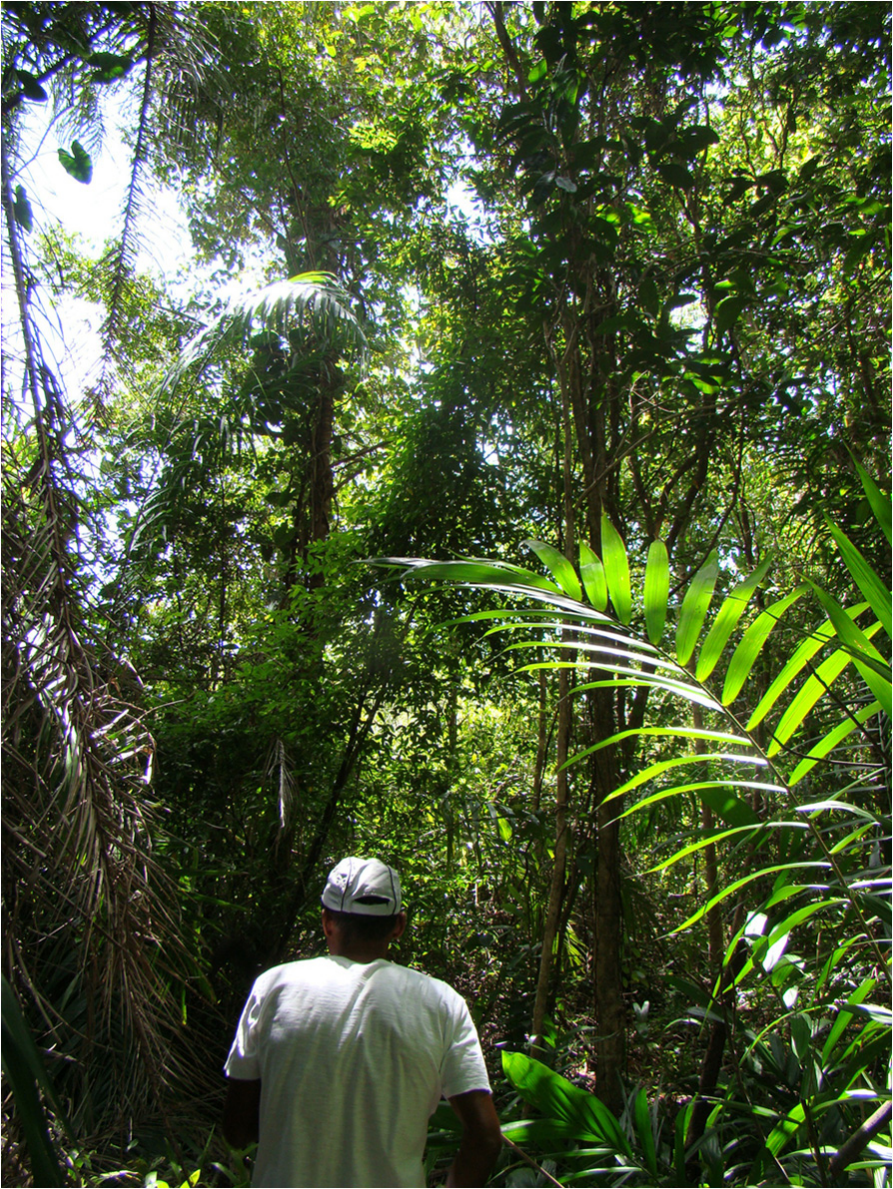
**An informant inside a *paú *** **area in the community of Ipiranga, state of Paraíba.**

The communities are located in Atlantic Rainforest region of Paraíba state, which over time has been replaced by large monocultures of sugar cane, a characteristic of the economic expansion of the region. When asked about the preference of the bees for a particular environment of the region, the informants were unanimous in indicating the *paús*, which are shady environments with remnants of trees, forming dense vegetation coverage and having an abundance of water (Figure 9).

When questioned about the sites of stingless bees nests, the informants reported that the bees make their nests in large trees, and did not mention any particular plant.

However, concerning the collection of food resources, the relationship between stingless bees and specific flowers, which are sought by the bees for honey production was emphasised. Thus, 17 categories of plants (Table 4) which, according to the informants, are the most visited ones by the stingless bees in the region, were defined. It is noteworthy that the beekeepers plant some of these trees in their crops.

**Table 4:** Most-visited plants by stingless bees, according to beekeepers from the communities of Ipiranga and Gurugi, state of Paraíba

**Family**	**Scientifc names**	**Local name**	**Habit**
Fabaceae	*Bowdichia virgilioides* Kunth	Sucupira	Arboreous
Fabaceae	*Mimosa caesalpiniifolia* Benth.	Sabiá	Arboreous
Myrtaceae	*Psidium guajava* L.	Goiabeira	Arboreous
Myrtaceae	*Psidium cattleianum* Sabine	Araça	Shrub
Anacardiaceae	*Tapirira guianensis* Aubl.	Cupiúba	Arboreous
---	*Non-identified*	Gobiraba	Arboreous
Myrtaceae	*Eugenia uniflora* L.	Pitangueira	Shrub
Rutaceae	*Citrus* sp.	Laranjeira	Arboreous
Bignoniaceae	*Handroanthus impetiginosus* (Mart. ex DC.) Mattos	Pau d’arco	Arboreous
Sapindaceae	*Talisia esculenta* (Cambess.) Radlk.	Pitomba	Arboreous
Fabaceae	*Apuleia leiocarpa* (Vogel) J.F.Macbr.	Jitaí	Arboreous
Clusiaceae	*Symphonia globulifera* L.f.	Gulandi	Arboreous
Anacardiaceae	*Spondias* sp. (Engl.) Engl.	Cajazeira	Arboreous
Anacardiaceae	*Mangifera indica* L.	Mangueira	Arboreous
Myrtaceae	*Syzygium sp.* Gaertn.	Jambo	Arboreous
Arecaceae	*Cocos nucifera* L.	Coqueiro	Arboreous
Anacardiaceae	*Anacardium ocidentale* L.	Cajueiro	Arboreous

### Symbolic constructions involving meliponiculture in the communities

The symbolic representations constructed around meliponiculture in the communities of Ipiranga and Gurugi are numerous. An intimate relationship between meliponiculture activity and such symbolic representations is present, mainly among elderly people, regarding beliefs and rituals. Such symbolic systems are understood here as learning tools, knowledge and communication among the group components, when a social integration and a construction of their cultural meanings are established [52].

The main ritual that is practiced concerns the collection of the honey produced by the *uruçu boca-de-renda*, when sexual abstinence is always practiced for three days before the day scheduled for collection. Thus, as soon as the collection is scheduled, both the beekeeper and other people involved in the process (men or women) are required to abstain from sex for three days prior to this date. According to the informants, if this requirement is not accomplished, the bees will bite the beekeeper and not allow the honey collection and, shortly after, the entire colony will migrate to another region.

Curiously, the practice of sexual abstinence is also noted among other groups of Afro culture, such as in the *maracatu de baque solto* or *maracatu rural* (a cultural manifestation of folk music) from Atlantic Rainforest region of Pernambuco state. The *caboclo de lança*, who is a character of the *maracatu rural*, practices sexual abstinence several days before carnival. This ritual involves the preparation of the group for the parade, when they perform in the streets of the cities [53].

There are temporal restrictions for females regarding the honey collection of *uruçu* bees, since a woman cannot approach the nest if she is in her menstrual or pre-menstrual tension period. Thus, keeping stingless bees is characterised as an almost exclusively male activity in the communities.

In *De Sangrias, Tabus e Poderes* (*Of Bleedings, Taboos and Powers)*, Sardenberg [54] suggests considering menstruation from a social and anthropological perspective and concludes that in many societies, menstruation is seen as a “polluting agent, gifted with impurities or a possessor of magical powers, generally evil”. Among many examples, the author cites the Ojibwas, who are natives of Canada involved in hunting, who consider the proximity of menstruating women as “dangerous and evil to that important activity for the subsistence of the group”.

The *uruçu* is considered by the informants as a *sacred bee*. According to them, *uruçu* bees “rezam o ofício” (pray) every Saturday and during the whole of May. Thus, during this period, they do not collect the honey from the nests from respect to the praying of the bees.

During study of the daily life of fishermen in the lower São Francisco in the state of Alagoas, Marques [33] observed the behaviour of those who “left the lagoon rest”, raising the question of the intentionality of such behaviour as an efficient conservation mechanism. The scientific field of ethnoconservation can be seen as a “new science of conservation”, which aims to meet both environmental and cultural needs and includes the traditional communities as “inborn allies in this exercise” [55].

The symbolic constructions reported here are embedded within a wider context that also covers the knowledge and the productive practices of the beekeepers. Only through the analysis of this group is it possible to understand the relationships between knowledge, interpretation and the management of natural resources by the communities in the construction of their worldview.

## Conclusions

The keeping of stingless bees in the communities is considered a traditional activity, which is involved in an ecological knowledge network constructed by different generations over time. The technique of making the *cortiço* characterized as the main traditional practice is orally transmitted from the generations over time.

The two communities share knowledge and similar management techniques well represented by the use of *cortiços* and the beliefs that underlie the creation of the *uruçu* bee.

The honey collected is a product of major importance in the medicinal tradition of the communities. It is largely used as a medicine to combat several diseases and is characterised as a product of “common use” amongst the residents of the communities, since it is always donated or exchanged, thus establishing a commercial relationship based on family unity and personal relationships.

A process of transformation has occurred in the meliponiculture activity of the communities, which is clear from the practices of the youth regarding elderly people. The use of rustic boxes to replace the *cortiços*, as well as the questioning of beliefs and rituals are characteristics performed by young people. These are seen, in most cases, as practices that unite (but not without conflict), the knowledge of the elderly and the new attitudes of the young people. Thus, a process of transformation and redefinition of meliponiculture practice has occurred, giving a cultural dynamic to the activity, as a demonstration that tradition is not static and is redefined in each generation.

Finally, the importance of biological and cultural diversity is emphasised here, in the study of the relationships between Brazilian Quilombola communities and beekeeping. These studies highlight the relationship between Quilombola communities residents and their beekeeping practices associated to the conservation of natural areas since these communities possess such knowledge.

## Competing interests

The author(s) declare that they have no competing interests.

## Authors’ contributions

RMAC carried out the field work, and all authors analysed and interpreted the data and drafted the manuscript. All authors read and approved the final manuscript.
